# The impact of yoga on occupational stress and wellbeing: exploring practitioners’ experiences

**DOI:** 10.3389/fpubh.2024.1352197

**Published:** 2024-06-05

**Authors:** Ingunn Hagen, Øivind Hagen

**Affiliations:** ^1^Department of Psychology, Norwegian University of Science and Technology (NTNU), Trondheim, Norway; ^2^Department of Leadership and Organizational Behavior, BI Norwegian Business School, Trondheim, Norway

**Keywords:** yoga, occupational yoga, yoga for coping with stress, yoga and wellbeing, yogic breathing, practitioners’ experiences, qualitative research

## Abstract

**Background:**

Workplace stress is a serious problem globally. It represents a major threat to the UN’s sustainability goal of good health and wellbeing (SDG 3). The purpose of this article is to explore how yoga may be a tool for increased wellbeing and stress management at work and in everyday life.

**Methods:**

To examine how yoga can facilitate employees’ wellbeing and ability to cope with stress, we performed qualitative interviews with practitioners who did yoga regularly. We focused on how yoga was experienced by each of our interviewees and what practicing yoga meant to them. Our data material consists of 13 semi-structured lifeworld interviews. The sample consisted of 10 female and 3 male in the age range of 20–55 years old. The data were analyzed through a thematic analysis.

**Results:**

The themes identified in the thematic analysis include: (1) yoga as a tool for increased wellbeing, (2) yoga for coping with stress and dealing with challenges, (3) the role of breathing, and (4) contextual factors. While confirming other research findings, this article elaborates on aspects informants described as induced by yoga, like self-awareness, calmness, balance, mood-lifting, focus, presence, self-care, and mastery. The reported positive outcomes of yoga constituted increased wellbeing, and also facilitated the ability to cope with stress and experience less stress. Informants also emphasized that yogic breathing was a central factor in inducing wellbeing and feeling less stressed. They also expressed that contextual factors, such as time, teacher, and location, influenced how practicing yoga was experienced and made sense of.

**Conclusion:**

The study concludes that the interviewees experienced practicing yoga as positive, by reducing their occupational stress. Moreover, yoga increased their wellbeing, as well as their ability to cope with stress. These experienced changes were especially facilitated by yogic breathing, and influenced by contextual factors.

## 1 Introduction

Workplace stress is a serious problem globally. In a recent Harvard Business Review report called *The Battle against Workplace Stress: How Smart Organizations Are Creating Healthier Environments* from 2023, it is emphasized that 82% of respondents said that workplace stress was a problem at their (US) organization. The same study found that stress in the workplace had a negative impact on employee engagement, creativity, and innovation while also contributing to employee turnover and burnout. Workplace stress was said to have a negative impact on collaboration, teamwork, productivity, and work quality. As much as 97% said that workplace stress could lead to mental health problems like anxiety and depression among employees. The consequences of occupational stress were devastating to both companies and employees. While many organizations were aware of the problem, fewer workplaces could make employees’ stress and mental health a priority. However, interest in dealing with work-related stressors improved slightly during the recent COVID-19 pandemic (1).

Studies have shown that also in Norwegian workplaces, stress was a problem due to the rapid changes and increased demands in many companies and the discrepancy between demands, expectations, and resources, which made it difficult for employees to cope (2, 3)^1^. Moreover, the borders between family and work have become increasingly permeable, as Hochschild (4) wrote about in her classic work, *The Time Bind: When Work Becomes Home and Home Becomes Work*. The borders between work and home have been further blurred by the massive shift to working from home during the COVID-19 pandemic, and frequent home office visits now seem to have become the new norm (5–7). There are also stressful experiences related to digitalization and technology in many workplaces^2^ (8). Occupational stress can be related to a rapidly changing society with increased complexity, global developments, and problems. Thus, large parts of the population experience their lives as increasingly challenging and stressful.

People who experience stress over time, in their jobs and/or in their everyday lives, can develop both mental and physical health problems. Examples of consequences of occupational stress include mental health problems like depression, anxiety, and burnout (9–11), physical health problems like back pain (12), cardiovascular health issues (13), as well as loss in productivity (14, 15). Yoga is about to become a complementary way to increase wellbeing and facilitate coping with stress for employees in contemporary society (16). In their article “Implications of Corporate Yoga: A Review,” Bhandari and his colleagues found that “CY [Corporate Yoga] is a cost-effective, eternal and universal means for workplace wellness and excellence that needs to be included as an indispensable part of corporate culture” (2012: 656).

In line with this, in the current article, we want to further explore the potential of yoga as a tool for coping with occupational stress. We acknowledge that yoga is an efficient way of dealing with occupational stress for many people. Here we aim to deepen the understanding of the positive relationship between, on the one side, yoga and, on the other side, occupational stress and wellbeing by examining how people practicing yoga themselves describe the connection between yoga, wellbeing, and stress. This can be summed up in the following research question: *How do people who practice yoga describe the impact yoga has on their wellbeing and on how they deal with occupational stress?*

## 2 Background and concepts

In the introduction, we described some current social tendencies that challenge people’s wellbeing and contribute to their everyday and occupational feelings of stress. The mentioned Harvard Business Review report, *The Battle against Workplace Stress*, points to how a high number of people today experience their work–life as quite stressful and challenging. One potential stressor is the way new media technologies have been integrated into our lives and work lives, together with a more commercial and competitive society. In the digital age, people are often overstimulated due to their availability through online communication and social media (17). New technological platforms and more mobile media are great resources, but they also make people available to others, including the workplace, 24/7. Workplace messages around the clock can make the boundaries between work and family life rather blurry. Constant online impulses and messages put people in continuous contact with the external world, potentially at the expense of contact with their own “inner world.” On the contrary, yoga can facilitate inner-world focus—on the breath, body, and mind—especially if practiced regularly (18).

Below, we aim for conceptual clarification related to some of the terms used in our title, “The impact of yoga on occupational stress and wellbeing: Exploring practitioners’ experiences.” This will provide background and perspectives for our study, whose purpose was to explore how yoga could be a tool to cope with stress and increase wellbeing in people’s work and everyday life.

### 2.1 Yoga

There has been an increasing interest in yoga in recent decades, which has been applied in different settings, including workplaces. Yoga is a Sanskrit word that means union or uniting, referring to the union of body, mind, and spirit. The often-quoted Sanskrit definition of yoga is “yogah chittavritti nirodhah,” the second sutra from the ancient, classical text Patanjali’s *Yoga* Sutras (see (19)). Thus, according to Bhavanani, the classical definition of yoga is “as a discipline to control the whirlpools of the subconscious/unconscious mind” ((19): 6). A general understanding is that yoga calms the fluctuations of the mind and thus creates stillness (see also (20)). However, there are many variations in the translation of this definition, such as “Yoga is the stilling of mental turbulence” ((21):16). According to WHO (22), yoga can be characterized as a traditional form of health promotion, and they recently described yoga as important in healing pain, including long-term back pain, and in facilitating relaxation. Yoga is an ancient Indian practice that often includes Asana (postures), Pranayama (breathing techniques), Dhyana (meditation), and relaxation techniques (12).

Holistic yoga is also seen as a way of life, or lifestyle (18). Moreover, yoga is also about getting to know oneself and thus facilitating personal development. The development achieved through yoga can, according to Gitananda (18), be described as a process of conscious evolution through creating a four- or five-fold awareness: developing awareness about the body, mind, emotions, meta-awareness, and awareness about one’s lack of awareness. Yoga can also be defined as “skill in action,” a kind of life mastery or resilience when facing challenges. Modern yoga is very diverse, varying from practices emphasizing asanas to traditions emphasizing meditation and mindfulness, basing themselves on one or several yogic traditions. While there are many yogic traditions, classical yoga is often based on Patanjali’s eight limbs (Ashtangas): Yamas (social restrictions), Niyamas (internal disciplines), Asana (physical postures), Pranayama (control of prana or life energy through breath), Pratyahara (withdrawal of the senses), Dharana (concentration), Dhyana (meditation), and Samadhi (union or integration) (23).

### 2.2 The concept of stress

There is also a need to understand stress better. Stress is a major source of health challenges, especially in the West. As indicated above, one of the contemporary challenges to people’s wellbeing is stress, and long-term stress is known to cause mental health problems like anxiety and depression (see (24)). Today, a lot of people experience stress daily, both at work and at home, as modern living is characterized by a high pace, constant performance pressure, and competition. Stress is something that affects the organism that is experiencing it (25, 26). The stress response is a set of physiological responses. When we experience stress, the sympathetic part of our autonomic nervous system is activated, and we experience a biological «alarm reaction», where our immediate impulses are fight, flight, or freeze. Thus, it is possible to define stress as an exaggerated response to changes in the environment, externally or internally (27). Stress is a condition that drains our energy, and typically long-term stress creates mental and psychosomatic health problems.

However, both the perception of and reactions to stress vary from person to person. It is important to recognize that one’s appraisal of a stressful situation determines one’s reaction to the situation—whether one allows the situation to affect oneself or not: “We may place our body on ‘stress alert’ quite unconsciously because of our psychological and emotional attitude” ((28), *Cyclopedia*, Vol. 3, p. 13). Thus, stress is based on our perception, not only the challenges of situations. In contemporary society, there is a growing concern with psychological wellbeing as a contrast to stress, fueled by the growth in the tradition of positive psychology. Thus, concepts like happiness, flourishing, and quality of life have become more central, together with wellbeing (see (29)). Wellbeing is often seen as a fundamental element of and precondition for good health, which is defined by the WHO as a state of complete physical, mental, and social wellbeing, not just the absence of illness^3^. Later, spiritual wellbeing will be added, too.

In numerous companies and workplaces, there has been a growing interest in yoga interventions for stress reduction and the promotion of mental and physical health. The reason is that yoga has many elements that can contribute to mental and physical health and wellbeing. However, there is a great variety of yoga practiced in the world today, varying from classical and holistic forms to yoga where the physical aspects are central. For example, in their study described in the article “The effects of yoga on stress and psychological health among employees,” Maddux et al. (30) used the term “gym yoga” for the yoga interventions they employed. This could illustrate a tendency in occupational yoga, as well as in yoga in the West, that the focus is primarily on the physical aspects of yoga. This is a reductionist perception of yoga, compared to the more classical versions (23). Reasons for the popularity of yoga in workplaces are that yoga is for all and can be performed regardless of age and previous knowledge, and that yoga is economical as it requires minimal equipment.

## 3 Research review and perspective

### 3.1 Yoga for reducing stress and increasing wellbeing?

In research on yoga, health and wellbeing are of interest, but coping with stress and stress reduction seem to be the main focus (31–36). How can yoga be a preventer of experiencing stress and a stress reliever? Several scientific articles show a significant reduction in the experiences of stress after practicing yoga (33–37). Thus, yoga seems to reduce stress levels or stress symptoms. In a review article, Sharma (35) claimed that yoga appears to be a promising modality for stress management. In another systematic review article, Chong et al. (33) concluded that yoga had positive effects on stress reduction in healthy adults.

Originally an ancient body–mind practice, yoga is currently regarded as an effective tool to promote mental and physical health and reduce stress (38). Moreover, several authors point to the need to better understand the long-term impact of stress as well as the underlying psychological mechanisms of stress (39, 40). In their meta-analysis of how yoga works, Ross and Thomas (41) claimed that the positive impact of yoga on mental and physical health can be explained by yoga downregulating the hypothalamic–pituitary–adrenal (HPA) axis and the sympathetic nervous system. The same explanations were emphasized by Pascoe and Bauer (38), who also found that yoga contributes to less depressive and anxiety-related symptoms as well as increasing the experience of calmness. Riley and Park (34) examined how yoga reduced stress through a systematic review of mechanisms for change. They found that positive affect, self-compassion, posterior hypothalamus, and salivary cortisol were all shown to mediate the relationship between yoga and stress.

Thus, what reduces stress seems to go hand in hand with what increases wellbeing. According to Li and Goldsmith (37), psychological mechanisms include a positive attitude toward stress, self-awareness, coping, feeling in control, calmness, mindfulness, spirituality, and compassion. Biological mechanisms included the autonomic nervous system and especially the relationship between the para-sympathetic and sympathetic nervous systems. Similarly, Riley and Park (34) found that the psychological mechanisms that relieve stress when doing yoga include a more positive attitude toward stress, self-awareness, improved coping mechanisms, more appraisal of control, increased calmness, mindfulness, spirituality, and (self) compassion.

There is an increasing focus on wellbeing and other positive outcomes, such as Verma et al. (12), who suggested that yoga can foster a sense of wellbeing, which will enhance relaxation and self-confidence. Related to wellbeing, it is important to mention that practicing yoga can facilitate relaxation and thus reduce experiences of and consequences of stress (24, 27). The increased wellbeing facilitated by practicing yoga is probably one of the reasons why yoga has become so popular in recent years, both in the West and other parts of the world.

### 3.2 Occupational yoga for reducing stress and increasing wellbeing?

Stress is assumed to impact people’s mental and physical health, as well as in the workplace, where there may be less efficiency due to burnout, sick leave, and high turnover. Thus, stress can be costly for both workplaces and society, something that motivates attempts and interventions to reduce stress and increase wellbeing. Naturally, workplaces with demanding tasks and stressful environments can have a negative impact on both mental and physical health. Stress is generally a response to a stimulus perceived as threatening, something that will activate the sympathetic part of the autonomic nervous system. Some stress can be positive, as it contributes to activation and may motivate employees to achieve desired goals (42).

Work-related stress is often related to the organizational level, such as heavy workload, the imbalance between resources and demands, conflicting demands, and frequent shift rotation (see text footnote 1) (43–45). Short-term and intense stress often have physical consequences, like headaches, muscle tension, flu-like symptoms, and increased heart rate and blood pressure (46). When perceived stress is prolonged, it can result in more serious consequences, like counterproductive behavior (14, 15), acute mental and physical health challenges (14, 47), burnout (45, 48), and a reduced sense of wellbeing (49). Thus, it is important to prevent workplace stress and to provide tools to counteract stress.

How has yoga been found to contribute positively to employees in the workplace? As indicated by Bhandari and his colleagues (50), yoga at the workplace can enhance people’s wellbeing and their performance at work as well. Much research on yoga in the workplace was done by Hartfiel and his colleagues (51), who found that people who practiced yoga experienced more meaning and satisfaction in life and had greater self-confidence when approaching stressful situations. Yoga in the workplace also contributed to stress reduction and improvement of back pain (52). Furthermore, in a study of workplace yoga interventions among British local government authorities, Hartfiel et al. (52) found reduced perceived stress and back pain and improved psychological wellbeing. In another study with university staff, Hartfiel and his colleagues (51) emphasized that yoga was effective in enhancing emotional wellbeing and resilience to stress in the workplace. The results further included marked improvements in feelings of clear-mindedness, composure, elation, energy, confidence, life purpose, and a feeling of greater self-confidence in stressful situations. Thus, yoga seemed to be a promising tool for stress reduction and increased wellbeing in different workplaces.

Experiencing workplace wellbeing is often related to the internal culture and organizational factors of a workplace, as well as to the employees’ own internal personal factors (53). It also matters what profession you have: For example, health professionals often have high levels of stress in their job and can benefit from yoga (43, 54). Also educational professionals experience stress due to contradictory demands, and find that yoga strengthens their ability to cope (12, 30, 44, 55). Wellbeing at work is important for all professions, considering the time and effort involved in tasks and performance, as well as the organizational outcome (56). Increased wellbeing can be a protective factor against mental health problems, as well as severe problems like anxiety and depression. Practicing yoga regularly has been found to result in high levels of wellbeing, psychological functioning, and happiness (14, 44, 57). Thus, workplaces need to develop health and wellbeing-promoting initiatives like yoga in the workplace to improve occupational wellbeing.

### 3.3 A salutogenetic perspective

A salutogenic perspective is relevant when discussing yoga, wellbeing, and coping with stress at work and in everyday life. The salutogenic model was developed as a theory to guide health promotion (58). Health promotion is the aim when using yoga to promote wellbeing and the ability to cope with stress in the workplace. The salutogenic perspective is a fruitful paradigm for health promotion, research, and practice, as it emphasizes facilitating health rather than just aiming to prevent disease (see (59)). From a salutogenic perspective, health and disease exist on a continuum, rather than being a dichotomous classification. Thus, health promotion must include all people, regardless of where they are on the health/disease continuum. The focus of Salutogenesis is on factors that contribute to health, rather than merely on factors lowering risk. Antonovsky (op. cit.) emphasized that health promotion needs to be concerned with the person, rather than his or her degree of health and disease.

A major concern in the Salutogenic orientation is how a person can be helped to gain greater health. Like in yoga research, there is a holistic understanding where the aim is to include all aspects of the person. According to Barry, when health promotion is based on a salutogenic view of health, it is “concerned with enabling individuals and populations to increase control over and improve their health and wellbeing” (2022: v) (60). There is also a shift from individuals at risk for developing illness to environments and systems, such as workplaces, that take responsibility for facilitating the development of good health for populations (like their employees). Sense of Coherence (SOC) is a central concept in the Salutogenic perspective, referring to perceiving the world as meaningful (motivates), comprehensible (challenges are understood), and manageable (resources for coping). SOC resembles other concepts of interest to us in our focus on yoga in the workplace, especially the notion of coping, but also optimism and self-efficacy.

The SOC concept is also central in the *Handbook of Salutogenesis* (59). Here, one of the main contributors asks the questions: “How may we better understand the origins of human health? How may we advance health in a manner considerate of the connectedness of health to life generally?” (61: 4). The answer, according to Antonovsky (58), is to assist people in developing a strong SOC. Thus, Salutogenesis is forwarded as a general approach to health theory, research, and practice, with emphasis on factors that facilitate health. The sense of coherence (SOC) concept is the main way to formulate what resources and factors people draw upon. The salutogenic perspective and SOC are compatible with the understanding of yoga as a method for human development (or self-realization, as Maslow would say (62)), increasing their awareness of strengthening factors that contribute to better health and wellbeing, and the ability to cope with the stress of life and work.

How can the research findings about yoga contributing to reduced occupational stress and increased wellbeing in the workplace be deepened by statements from our interviewees practicing yoga? And can the Salutogenic perspective of health as a continuum provide a framework for their perception of health, wellbeing, and stress?

## 4 Methodology

### 4.1 Context of the study

The research project that this article is based on is called “Yoga, wellbeing and coping with stress in work-life and everyday life.” It was performed in Norway, which, together with the rest of the region of Scandinavia, is regarded as leading in work–life balance (63, 64). However, work-related stress is a threat to a healthy work–life balance in Scandinavian work–life too, as well as globally^4^.

This research project was organized as a research practice for bachelor students, with the first author as the initiator and project leader. In addition, two master students assisted in all phases of the research project to facilitate the quality of the work. The purpose of this study was to explore how yoga may be a tool for coping with stress and contributing to increased wellbeing for employees who practice yoga regularly.

### 4.2 Data material: the interviewees

Qualitative interviews were the chosen methodology, as the aim was to understand how these employees experienced practicing yoga, and the impact they felt that it had on them. Recruitment of informants was done through companies that offered yoga to employees and through yoga instructors. About half of the informants were recruited through “a snowball methodology,” where we recruited informants via acquaintances who practiced yoga, or through the already interviewed informants.

The interviewees with information like fictive names, sex, occupation, age range, place of performing yoga, and time of practicing yoga are presented in Table 1.

**Table 1:** Overview of the interviewed yoga practitioners in our study.

Fictive name	Sex	Occupation	Age ran e	Place of yoga	Time, yoga
Randi	Female	Researcher	35–45	Workplace	1 year
Kari	Female	Researcher	40–50	Workplace	6 years
Katja	Female	Researcher	35–45	Workplace	2 ears
Inger	Female	Researcher	40–50	Workplace	2 years
Bernt	Male	Engineer	25–35	Work, home	3 years
Marit	Female	Manager	40–50	Work, home	2 years
Sonja	Female	Graphic designer	25–35	Home	5 ears
Anna	Female	Civil economist	25–35	Work, home	1 year
Martin	Male	Civil economist	25–35	Home	2 years
Liv	Female	Grade leader, Rima School	25–35	Work, gym	6 years
Anja	Female	Adviser	45–55	Gym	2 years
Harry	Male	Hairdresser and taxi driver	25–35	Gym	Not known
Linda	Female	Receptionist, gym instructor, and student	20–30	Gym	Not known

The table above presents the 13 interviewed yoga practitioners, who consisted of 10 female and 3 male in the age range of early twenties to mid-fifties, from a diverse range of professions. The informants were all Norwegian. The majority of them had higher education. The interviewees had different experiences with yoga. Eight of them participated in yoga at their workplace, two mainly practiced yoga at home, and three of them practiced yoga only at a gym. Among the eight who participated in occupational yoga, one also did yoga at a gym, and several of them also practiced yoga at home to various degrees. The participants varied in the length of their yoga practice, between 1 and 6 years. All the interviewees were working. The youngest interviewee was a student as well.

Our data material consisted of so-called *lifeworld interviews* (see (65, 66)) with employees from a variety of workplaces. Lifeworld interviews are suitable when the goal is to understand phenomena (such as practicing yoga) in the everyday life of interviewees from their own perspectives. The purpose of this kind of interview is to understand the lifeworld of the interviewees and to interpret the meaning of the described phenomenon. The advantage of this kind of qualitative interview is that the interviewer uses an interview guide in a flexible manner, where the wording and order of questions vary, and allows for follow-up (probing) on important and interesting answers and themes in the interview situation. We also did a background interview with a yoga instructor who taught yoga in both workplaces and in a yoga studio.

### 4.3 Data gathering

The interviewers in this study were all BA students, who got continuous support from the project leader and two MA student assistants. The teaching they took part in and the continued follow-up on the project seemed to secure good quality of the interviews. In the interviews, the interviewees were informed about the purpose of the project and that they would be secured in terms of anonymity, both through the way the data were stored and the way the results were presented. We applied to NSD.no (now Sikt.no), the Norwegian council, for approval of research and were granted permission to do this study. Only those who were part of the project had access to the data, and they were stored properly. Anonymity was secured by using fictional names and by describing the age range rather than the exact age. The interview guide used ensured that all the interviewees were asked similar questions, but not necessarily in the same order or formulated in the same way.

All the interviews were performed face-to-face, with two interviewees present. One interviewer was responsible for asking questions, while the other took notes and audiotaped the interview. Both the interviewers asked probing questions during the interview. At the beginning of the interview, the interviewees would be informed, orally and by a written statement, about the background and purpose of the project, what participation implied for the interviewees, how the data were stored and used, that participation was voluntary, and that they could withdraw at any point. The interviewees signed a voluntary, informed consent form. All the participants also agreed that the interviews were audiotaped. After the information and framing of the interview, the interviewees were asked to talk about their everyday work experiences, including stress at work and in their everyday lives. They were also asked about their experiences with yoga, about yoga in their workplace, and the potential consequences they experienced related to practicing yoga. Then, the interviewer presented a summary of the interview and checked it with the interviewee. Finally, interviewees were asked if they had anything more to add.

### 4.4 Analyzing the data

The data were analyzed through thematic analysis (cf. (67–69); see also (70)) to get an overview of dominant and important themes in the data material. Thematic analysis is about comparing and contrasting patterns within and across interviews. The interviewees’ experiences were emphasized, focusing on the semantic expressions. The procedure for thematic analysis described by Braun and Clarke (67) was generally followed: (1) getting to know the data, (2) generation of initial codes, (3) using the initial codes in identifying and searching for themes, (4) developing and reviewing of themes, (5) defining and naming of main themes, and (6) writing up the results in an article or report. The main themes identified included mental health and wellbeing, yoga and stress, the role of breathing, and the contextual aspects experienced related to practicing yoga. The interviewees were interviewed in their mother tongue, Norwegian, transcribed ad verbatim, and analyzed based on the Norwegian transcripts. The chosen citations have been translated into English for the current study.

### 4.5 Critical reflections and possible limitations of the study

The undergraduate students performing the interviews may be criticized for being inexperienced. As discussed, we were aware of this limitation, and the fresh interviewers were followed up and guided by graduate students and the first author as part of the project and the students’ training. In addition, the interviews were performed using a clear but flexible procedure involving, among other things, a summary of the interview that was submitted to the interviewed. Our impression of the interview data is that the quality was good and that the interviews generated valid data on the research topic.

The sample of interviews was based on convenience logic (71). As discussed, half of the informants were picked from companies that offered yoga to their employees, while the other half was picked on a snowball logic, meaning that the first group of informants came up with suggestions of people doing yoga via their employers. This may have led to a biased sample, and it represents a threat to the representativeness of the study. Still, in qualitative studies, the aim is to get a deeper understanding of a phenomenon, like corporate yoga, and not to get representative results statistically (72). Due to the logic of qualitative research, it is necessary to redefine concepts for discussing the quality of (quantitative) research projects, such as validity, reliability, and generalizability (see (73)). Validity is often replaced with a process of constant validation, reliability is about creating credibility through being systematic and transparent, and generalizability is often discussed as transferability—to what extent results can be used to explain or understand similar phenomena or contexts (65, 70). We have tried to live up to these ideals by critically reflecting on the quality of the data, analyses, and arguments, by openly describing the steps of the research process, and by giving context to both the project and the interviews.

## 5 Main results and discussion

The main results from this study tell us that all 13 interviewees experienced yoga as contributing to increased mental and physical health and wellbeing and enabling them to cope with and experience less stress. These findings reinforce what other research has found, both about the impact of yoga generally on stress and wellbeing (33–38) and about the beneficial impact of yoga related to workplace stress (Bandari et al., 2012; (43–45, 51, 52, 56, 74, 75)). These positive impacts of yoga seemed to have been especially facilitated by yogic breathing or Pranayama. The impact of practicing yoga was also influenced by contextual factors, like the time of doing yoga, the relationship with the teacher, and location.

### 5.1 Yoga and wellbeing

All the informants expressed that practicing yoga gave them a general feeling of wellbeing. Many informants described that they looked forward to the yoga practice and that they experienced satisfaction after class. Most informants said that they felt better when they practiced yoga.

The informants revealed that they felt that practicing yoga was good for them, both as experienced and anticipated, something that seemed to motivate them to continue. They also emphasized that yoga impacted their whole everyday life, both at work and beyond:

*“I feel that it [yoga] gives an energy boost. Very good in the body afterward, so it also provides well-being.”* Bernt

Similarly, another interviewee found what happened in her body during yoga difficult to explain, but described a bodily change that made her feel more lighthearted and less heavy in the head.

*That’s where I think that it’s not something you understand until you do a yoga practice, it’s just something that happens inside the body, which you cannot quite explain—because it’s not so easy to understand—it’s just something that has let go a little, and is a little more light-hearted, and there is something in my head that does not feel so heavy anymore.* Anna

The above informant also described how yoga helped her let go.

Another informant expressed that she also felt more positive after yoga, as her to-do list, which made her stressed, decreased in significance. This improved her wellbeing at work.

*I often feel … a little more… positive! The thoughts are more positive than before, it has a lot to do with the fact that before I thought about a lot of different little things I had to do and felt a bit stressed and afterward it’s gone and I feel like “Well, I can have a good day at work! “I can do a lot, so….* Katja

Informants also expressed that practicing yoga contributed to more awareness about their feelings, energy level, mind/thoughts, and ability for self-care. A female and a male informant elaborated:

*“I feel I am becoming a little more aware. I notice from day to day how I feel… Whether I feel tired or have a lot of energy, but also mentally, where my thoughts fly and… Yes.”* Sonja

*“I have become better at taking care of myself, I think. And looking after my own interests. Before, I would never have thought that it would be possible to work reduced hours, for example. I have always been very conscientious. “In any case, I have to work 100%, and maybe more.” But now I know that it is my life….”* Bernt

The above informants had gained an awareness of themselves and their inner world, something that gave them more agency, and the ability to make good choices.

Similarly, one of the interviewees expressed that practicing yoga facilitated his self-compassion in the sense of being kinder to himself by paying more attention to himself and his body’s needs:

*“Also, I’m a lot kinder to myself, maybe, I do not know.”* Harry

Interviewer*: How about that, be kind to yourself?*

*“Both in the way of eating and… maybe that and… that you… go to bed earlier, get up earlier? If I’m a bit tired, then… I might rather choose to go home and relax, and rather get ready for the next day. Instead of burning the fuse … the day before.”* Harry

In the same manner, a female informant described that she became more aware of her limitations and thus kinder to her body, as doing yoga reacquainted her with her body.

*“…I know my limitations better now, and I actually feel more confident that I’m kind to my body… It’s quite fascinating to have lived in your own body for so long… and then at the age of more than 20 suddenly almost get to know it again… I think it’s absolutely crazy….”* Liv

A similar shift from primarily being influenced by the external world to getting more in touch with their inner selves was also expressed by others, like the male informant below:

*“Often the focus is on the outside, on everything external. And you look at everything around you. Then the focus changes a bit. And maybe that’s what it is… What is happening then…. When I’m good, I’m focused inward, while when I’m not as good, I’m focused outward… And there’s a kind of learning in that and then, and turn the focus a little inward, and become comfortable with that. Thus, yoga is probably one of those things that allows you to, can help you a little to work on yourself then.”* Martin

It seemed that this informant, like several others, felt that the shift toward more internal focus was beneficial for him, as he could work on himself and develop as a person. It is also interesting that he described that he had to learn to become comfortable with his inner focus.

Another quote about the experience of getting in touch with oneself came from a female informant, who explained that she shut out everything else and turned her focus inward during yoga:

*“I can focus very well on myself, sometimes I also close my eyes and just go into my own little world, into my body, and feel ‘what’s going on here?*’” Katja

Seemingly, this ability to focus on herself and the practices facilitated the ability to concentrate. The same informant expressed how yoga made her more efficient and focused:

*“More often things go better, more often I am more efficient after yoga. I am more focused on what I really have to do.”* Katja

Another interviewee also expressed that yoga affected his work performance positively:

*“Now it’s one hour a week, so it’s a very small part of my life. But I feel that it is helping to move in the right direction. It gives me something that enables me to perform better at work. I think so. And especially that presence. Being present in the body, and in the silence. Often that’s where the good ideas come from.”* Bernt

This bodily presence was reported to be experienced as very useful and positive:

*“Yes, so after a day at work, it is very good to reconnect with your body. I’ve been in my head a lot. I notice that. Thus, in the yoga class, when the focus is on breathing, on keeping the energy in the body, and on the exercises, it gives energy, I know. And that makes me more present in the evening. It’s a very nice ending to the working day, getting out of your mind and into your body. Into presence.”* Bernt

Yoga enabled reconnecting with the body, after having been too mentally engaged at work. Such reconnection was interpreted as facilitating energy and presence.

Several informants expressed that yoga made them stronger mentally by challenging themselves with difficult bodily positions and building a sense of mastery that could stay with them beyond the yoga class. In the words of Sonja:

*“I think I’ve also become a bit tougher from yoga then… It might seem a bit silly but… Just standing on your head, for example, was completely unthinkable for me four years ago, and only that I had the courage to work towards the exercises that are a bit scary then, it gives you such a feeling of mastery that I think you take with you into everyday work!”*

Another interviewee, Anna, expressed that she used yoga to deal with a difficult life situation caused by being harassed by a manager in her previous job. She described that she lost self-confidence and self-esteem and became depressed. She was able to reduce the notice period, got off a few weeks before starting a new job, and enrolled in a yoga studio for daily practice:

*“…I needed to go somewhere where I could… erm… calm down and get rid of these bad thoughts and feelings… (…) And that… yes, it actually helped me through that period. It is a concrete example of yoga helping me through a very painful and difficult period, for exactly this sense of mastery, thus helping me to build self-confidence, and a sense of mastery again.”* Anna

In addition, this informant experienced a sense of mastery, and she expressed that she became calmer, more balanced, and able to regulate her emotions after practicing yoga. In her own words:

*“I am calmer and more balanced after a yoga class, and that I have more control over my emotions. This applies to both the ones I have at work and the ones I have at home.”* Anna

Many of the interviewees expressed that practicing yoga made it easier to deal with psychological problems or difficult life situations. One female informant told that she had used yoga to rebuild herself after experiencing burnout:

*“But it is clear that… I have consciously used yoga, for some time, because of… ehh… ehh, because… a few years ago I was really burnt out, I had to take a break… for six months… from work. I had to rebuild myself.”* Anja

One of the male interviewees revealed that his partner had incurable cancer and had died about a year before the interview took place. This informant explained that he also used yoga to regain strength, also during the period when his partner was ill.

*“When my partner was ill, there were periods when I almost cut back on all my own activities, but to get through that time I needed something to replenish. And yoga was one of those things that I could not cut out.”* Bernt

The informants described many aspects that were part of their wellbeing experience: yoga increased awareness, balance, calm, more inside focus and self-knowledge, was good for them, gave an energy boost, was more lighthearted, less heavy, kinder to self, more self-care, more in control, more focused, increased presence, reconnected with the body, stronger, sense of mastery, self-confidence, and being rebuilt. Such increased internal focus, self-awareness, balance, and calm are often emphasized as qualities that yoga contributes to Gitananda and Kishida et al. (18, 76). Yoga also increased positive affect, energy, feeling in control, and self-care (34). Yoga as a skill in action could also be seen as a stronger sense of mastery and self-confidence (18, 51). Wellbeing also relates to increased psychological functioning, like increased focus and presence, feeling in control, and more content (14, 44, 57).

### 5.2 Yoga and stress

There is a lot of pressure in contemporary society, including a rather stressful work–life (77). There are also expectations of being able to perform well and perceived pressure to achieve success in all areas of life (78, 79). This perceived pressure can lead to experienced stress, which can result in physical pain as well as mental stress (51, 52, 75). However, stress is an experience that also depends on a person’s perception (28). How you experience the situation, what resources you have, and how you handle the situation mean that you can have different thresholds for what is experienced as stress. It should also be pointed out that some pressure or stress can have a motivating effect, but too much stress or stress over time can be problematic. This personal take on stress was pointed out in our study:

*“Thus, it can be stressful, but it depends on who you are, I think, and how much you stress about things. I’m maybe a bit of the type that I take small signs a bit inwards in a way, ehm….”* Kari

Another interviewee also shared that he regularly practiced meditation and expressed that both (physical) yoga and meditation helped to reduce stress.

*“The way I experience yoga, including meditation, is… [long silence]. A way to take a step back, out of the noise of thought. And I often feel that when I’m stressed, there are a lot of thoughts, ruminations, brooding. And in that sense, I feel that what yoga gives me is that I focus on something other than my thoughts. I focus on the breath, the body, the movements, the balance. And then there will be less attention to the stress, to the train of thought. In that way, I feel that it helps a lot.”* Bernt

Here, the meditative side of yoga created space for focus and greater awareness of bodily experiences, emotions, and thoughts (cf. (18, 80)).

Several informants mentioned the role social media and other media played in experiencing stress. Feeling that they always had to be available, up to date, with little time to be in their “own world” was perceived as stressful:

*“Eh, the series is on the TV over there, but I can quickly sit with a computer next to me, and suddenly an email or something like that pops up, you also get stressed because… it’s something school-related or work-related then. But in yoga, it’s like away from the outside world and all the technology and things like that. Eh, and it’s very nice. And do not think about the… technology part, you only have yourself to focus on, nothing else.”* Linda

Yoga was experienced as helping this interviewee and others to disconnect from the outside world and get more in touch with themselves after hectic working days and an everyday life filled with stimuli from various media technologies. Yoga was bringing more positive inner focus.

Thus, yoga seemed to be perceived as a sanctuary or refuge from external pressure, a place where you can disconnect and be with yourself. One interviewee explained that these yogic experiences stayed with him as a feeling of being more satisfied with himself and life, rather than chasing after something more:

*“But then something has happened to the body in that half hour, which means that you have a surplus at least for the rest of that day, and maybe for a few days afterward as well, and then you are happier and more satisfied, and think more about what you have, and not what you should have, and constantly spinning about what you want and how others feel, and how I should be.”* Martin

Similarly, a female interviewee expressed that she experienced yoga as a refuge from social expectations:

*“It requires a little bit from everyone on every occasion. When you are at work, you must be “such and such,” when you are with friends, you must be “such and such”; and something is expected at home if you have children, and there are always expectations of you then. And sometimes it can be very good to have a place where nothing is expected of you, and that is yoga.* Anna

Another interviewee described how yoga could help her release these destructive thoughts and provide calmness, presence, and contentment with herself.

*“Yoga becomes like a tool to cleanse the body. A tool to reduce stress and restlessness in the body, and definitely a feeling of mastery, to the extent that when I have peace in my head, and peace in my body, I can be myself, and then I am more satisfied with how I act in front of other people and the environment.”* Anna

It appeared that yoga for this informant was a way to let go of bodily and mental stress, which allowed for mastery, contentment, and being able to be your authentic self.

Perceived stress can also result in physical symptoms, such as pain or stiffness in the back and shoulders. This contributes to more stress, both directly in the form of discomfort and indirectly in the form of worry and anxiety, because you will not be able to do what you need to:

*“If I’m really stressed, there are some physical effects (….), I do not know, stiffness in my back and… various such… moments of irritation then in my body without being able to put my finger on what, what exact symptoms. “*Inger

Another informant experienced stress and bodily tension related to her doctorate work:

*“There is often a little tension in the muscles, but it also has to do with, since the Ph.D., there was a lot more to do there, but it still comes back, but I try to do yoga [laughs] or go for a massage or go out, and go out for a walk, so since my Ph.D. I have learned many ways to fight stress or yes… Work against stress… to… yes become a little less stressed.”* Katja

*“Sometimes you think about little things like that you have to do and I must not forget that I have to do this and that. And after yoga, it’s like “ahh”… “What else was I thinking about?” It’s not important anymore, you have much more peace within yourself and it’s important to me that I get it at least once every week.”* Katja

Among other things, this female interviewee actively used yoga both to loosen physical discomfort and to reduce perceived stress. This example illustrated how yoga was experienced to reduce physical discomfort and tension and thus calmed down a busy mind and stressful thought processes.

Several of the informants realized that their stress level was related to how they perceived situations, and for some, this changed with practicing yoga (cf. (28)). Some of the interviewees also described how social and other media were a source of stress by making them constantly available, both socially and in the workplace (17). They also used media for everyday relaxation but found that it did not always relieve the stress and tension they experienced after work, as well as yoga did (24, 27). In addition, expectations from others created stress, and yoga was perceived as relieving such stress. Indeed, yoga was perceived as a tool for reducing stress and bodily tension, as well as mental tension, as has been pointed out in the literature (34–37, 39, 40, 75).

### 5.3 The role of breathing techniques (pranayama)

Many of the interviewees emphasized how focusing on breathing often made them feel less stressed. Indeed, yogic “breathing techniques” (Pranayama) are often portrayed as a link between the mental and the physical, and breathing techniques can be used to manage and reduce stress (24).

Several informants, such as the interviewee below, described that they used breathing to calm down stress:

*“Then we sort of start with stress management, and that’s something I try to take with me further then, out of class.”*

Interviewer: *“Okay. In which situation then?”*

*“No, all the time when you are stupid enough to breathe, what can I say, only chest breathing. When you, what shall I say, get a bit too busy and there is a bit too much fuzz around you, you forget to, what shall I say, take the time to breathe.”* Harry

Another female informant described using breathing to calm down and be more mentally present:

*“… and on a working day where you go from one meeting to another, I can kind of come to the office and sit down, and kind of close my eyes and do some simple breathing exercises like that to kind of find calmness… yes, it actually works… yes, then you get it here… eeh… that you train an ability to log in a bit, a mini-mindfulness like that… which you can, which you get a lot of effects… from.”* Anja

Some interviewees, like the one below, found it helpful to use breathing to calm performance anxiety:

*“Yes, I try to use the breathing techniques before I have a performance, for example. If it is suitable… Hehe! I notice that I can manage to calm down… Easier than before! So I do maybe 10 breaths before a performance, so I’ll be more calm then.”* Sonja

Another informant expressed that it made her feel safe, knowing that she can do yogic breathing (and sometimes asanas) to seek refuge from stress.

*“It’s something I take care to do at work, for example. Just breathe, take a deep breath, and maybe also the exercises you do, and just stretch yourself a little, and… Just knowing that if things get a little overwhelming, you have somewhere to go, and that’s into a yoga—practice. And escaping a little from reality into it then. So, when I do these breathing exercises at work, I get more peaceful, and I use a bit of what I’ve learned in yoga practice by closing my eyes a bit, it’s a small form of meditation, and closing your eyes, telling yourself that it’s going well.”* Anna

Based on the statement of this interviewee, it can be interpreted that she felt that yogic breathing and positive self-talk enabled her to stay in and get through stressful situations.

For some interviewees, practicing yogic breathing was actively used to deal with stress and regain focus and calm:

*“It’s a simple example, if you are stressed you should, I was about to say something, but I think “No, first I’ll take a breath and then I can start talking,” then you become more relaxed and calmer, and stress… I think the stress go away and then you can… If you have some time inside your head, and when you become a little calmer then… Then maybe you will say what you intended.”* Randi

One female interviewee expressed that focusing on her breathing gave her energy, both during the working day and in other parts of her life:

*And it is exactly this one, that you get to train yourself to both use your breath…. in a different way… and to train yourself to mentally switch off.… eh, thoughts and what you are actually going to do, that that comes… what lies before you in a working day or… you have been through or. And doing that combined with these flowing movements then… which require quite a lot of concentration, it has given an incredible amount of surplus and energy.* Anja

Experiencing that she benefitted from yoga motivated another female interviewee to find time for practicing. After yoga, she felt calm and changed perspective, realizing that what kept her busy was less important:

*“And for me, yoga has become a priority, you could say. I know it’s good for me… I know I must make time… and if I stress about thinking I do not have time… Then I know it’s even more important to do it. Maybe I will not achieve everything I’ve set out to do… but after a yoga class, my head goes into a different mode, and then I think a bit like “Yeah, maybe it wasn’t important to achieve everything else today, why did I stress so much? It’s much better for me… to sort of take some time to just relax and practice yoga… it’s more important than maybe having time to buy…. yes you know… having time to buy washing powder before the shop closes….”* Liv

As the same informant pointed out, yogic breathing seemed to synchronize the mind and body:

*“There is something about the head and body being “tuned” to the same notch. Both are either relaxed, or both are working to do the physical, or both are breathing. It’s not like the head thinks about something, and the body does its own thing.”* Liv

Some participants seemed more motivated by the potential physical effects of yoga, such as becoming strong and flexible, while others paid more attention to the potential mental impact. For most of them, yogic breathing was appreciated, as it facilitated unity of body and mind (18, 76, 81). It seemed that yogic breathing had become a specific tool that the informants had learned to use for stress management, to both increase calm and focus, to be ready to perform, and to find focus rather than being stressed. In other words, yogic breathing was used to change mode from a sympathetic-dominated mode to a more para-sympathetic mode (38, 41).

### 5.4 Context for doing yoga: time, teacher, and location

The participants in our study also talked about the significance of the time they did yoga, the meaning and role of the yoga instructor or teacher, and also the importance of the location where they practiced yoga: at their workplace, at home, at a gym, or in a yoga studio.

#### 5.4.1 Time

The interviewees described practicing yoga at different times of the day with different benefits. Most of them did yoga in the morning, others in the afternoon or evening. One informant described how doing yoga in the morning gave a good start to the workday:

*“It will be a slightly different start to the day then. Thus, I would have liked to have done it [practiced yoga] there every day, but it does not work in practice. So, I feel that… I feel calmer and perhaps a little more focused, and ready for a proper start then. It’s a good start anyway.”* Marit

Another informant expressed that she preferred to do yoga in the morning, but experienced being reenergized doing yoga after work, although she had started out tired:

*“I think it’s best in the morning because then you kind of get that boost throughout the day. But I’ve done it a few times right after work too then…erm. and then I feel that even though I’m quite tired after a working day, and then I think like “puh, can I take this now”…"I’ll do it »… and I decide to do it anyway, and then I feel that when I go home from work afterwards I have gained new energy, so it is always…eeh… that you come out of it positively.”* Anja

One interviewee described how doing yoga after work could be destressing and increase awareness in the body, which may also benefit you the next day:

*“And it’s good to take a yoga session after work because then you might get to know a little about how you feel. If I’ve had a bad day, I might become aware that “Oh, now my shoulders are a bit high here. And now ‘Liv’, now you do not breathe with your stomach.” So, then you de-stress. You do not always know you are stressed either, so you get to confront your body right away. It also makes me more aware the next day. Then I can suddenly stop, and just ‘now I have that tight feeling like yesterday, why?’ and then maybe I take a breath for two seconds before I move on.”* Liv

#### 5.4.2 Yoga teacher or instructor

Numerous interviewees highlighted the importance of the teacher for the experience of participating in yoga. For some, the instructor could be decisive in whether they wanted to participate in the yoga class, depending on the pace that the teacher offered:

*“But it’s actually a bit like that I sign up for the class that suits my mood a bit, and then the instructor is often important… like that… today I feel like I’m being teased a bit by her… or no, today I go for it the hour where I know he/she runs a quieter schedule.”* Liv

In addition, personal feelings about the yoga instructor or trust in him/her mattered:

*"And then I think that it has a lot to say whether I like the instructor or not! Because some instructors are… if I trust them, I want to attend the class.”* Anna

The teacher seemed to be important in motivating the interviewees to participate, to carry out, and to push themselves to stay in uncomfortable positions. One interviewee pointed out that she needed to have an instructor to do yoga, who could guide her and motivate her:

*“It is very useful that there is an instructor who tells you what to do, guides you and not least tells you how long to keep on like now you have to stand in this uncomfortable position until I say stop. I would never have done that on my own initiative. You can, people say you can do exercises in your office, for example, but you cannot do it, and you can do yoga at home, but I cannot do that either.”* Inger

The fact that the teacher showed understanding, interest, and empathy was emphasized:

*“It is enough that you get to know each other a little, and… You go there for a little longer, and that the yoga teacher is interested in understanding you, and getting to know your body, which enables them to provide the right adjustments so that no damage occurs. And then it is very important that a yoga teacher has some empathy! And understanding that people have their lives, and maybe have a bit of a hard time for a while… Things like that….”* Sonja

Another female informant attended yoga at the company she worked for but previously went to a yoga studio during a period when she was recovering after a hard time in her life. Thus, she highlighted that the yoga teacher showed interest and was caring, especially when she felt vulnerable.

*“They must be caring perhaps! I think at [former yoga studio] they have been very conscious of who they have hired to lead the classes. Lots of good, caring people who SEE you, and they were very good at asking follow-up questions; “How’s it going today?” and “How are you today,” so it was very nice to be there. And I think I have a lot to say in favor of that, especially when I was doing it for a kind of repair, rather than prevention.”* Anna

For one interviewee, the instructor led her to stop participating at that particular yoga studio:

*“I have also experienced that my yoga teacher has said that it would not be prudent to teach me if I did not come every day…. So, it was all or nothing. And it was during a period when I had a lot of problems with sleep and a lot at work… So that was actually the last time I came to the yoga studio, and that’s when I decided… that… now I need to do this on my own! You have enough challenges in your everyday life, so you do not need to come there to hear that sort of thing….”* Sonja

The above informant was disappointed to be met with what she perceived as unreasonable demands and little understanding from the instructor. However, yoga was not dropped:

*“I think it’s just important not to stop, but to continue with yoga, and just adjust it according to how you feel then! Because yoga is a lot about paying attention to yourself and learning what you need. And do not be controlled by those who do yoga around, or yoga teachers who are too strict.”* Sonja

It seemed that this interviewee dismissed what she perceived as an unreasonable yoga teacher. Since she was aware of her needs, she chose to leave the yoga studio, but she did not stop doing yoga.

#### 5.4.3 Location

The interviewees varied in where they practiced yoga. Four participants only participated in yoga at their workplace, two only did yoga at home, and three only attended yoga classes at a fitness center. Three participants did yoga both at work and at home, while one did yoga at work and a fitness center. There were often practical reasons why participants chose to do yoga. The participants had various offers from the workplace, some had yoga during working hours, and after work (without pay), some had expenses (course fees) covered by their employer, others paid a part, while some employees covered the expenses themselves.

Some informants highlighted the accessibility of being able to do yoga in the workplace:

*“It is also very advantageous that it is at the workplace. It’s such a short way. Right after work, just change and go downstairs and join in for an hour. The door sill is very short. If it is that I had to go into the city and sign up for a class, then the threshold would have been higher. Thus, it is a huge advantage that it is at work.”* Bernt

*“Because it is offered here… and… that we get paid hours to do it.”* Katja

*“The threshold was slightly lower when it was during working hours than if I should have signed up. I would never have signed up for yoga at a fitness center because it is so far from what I usually do. But here, when it’s at my job, with people I know, during working hours, it’s much, much easier.”* Inger

Yoga being offered during working hours, with colleagues, and not having to travel anywhere, was appreciated.

Several of those who participated in yoga at their workplace pointed out that they felt grateful to the workplace for offering yoga at the company:

*“What I feel perhaps the most is gratitude for the fact that we get to have that yoga class there. So that makes me want to do a good job. And I feel very looked after, in that, we get to have it, yoga at work.”* Bernt

*“I have no feeling that the time I spend on yoga eats up time that I would have been productive, in fact not at all. Rather the opposite is that I am so happy that I am allowed to spend this time on yoga that I work better because of it.”* Inger

It is interesting to notice the sense of gratitude that informants expressed for being offered yoga at the workplace; it made them want to give back to the workplace by doing their best.

Several informants expressed that yoga made them less stressed, more aware of themselves, and more patient with other people, as well as at their workplace.

*“I also think that I have become rounder at the edges. I can still stand my ground and believe that my reality is the most real. But I have probably become a little more rounded, in that I also see that it is not only what is real. Everyone has their reality. To a greater extent than before.”* Bernt

*“I think I’ve become a little more relaxed, I feel it myself at least… I feel better around other people… it’s almost like it makes me a little more patient….”* Liv

*“I am perhaps a bit more stable in my mood. That you do not get irritated so quickly, or, it’s very difficult to say. (…) but then, you might manage not to… get fired up, just as quickly.”* Anja

*“You become more positive. You become more relaxed. You become calmer… and that’s part of relaxed, after all. I’ll be… I’ll be much more pleasant… like that if you know what I mean. A bit like that will be a bit calmer and saner, I think. Instead of being a bit abrupt and quick, it may be that I become a bit more relaxed.* Harry

All the quotes above showed how the informants felt they had become more open to other people’s perspectives, more relaxed, calm, and patient, something that was regarded as positive for cooperation with others and for the working environment.

## 6 Discussion and conclusory remarks

In this study, we focused on how yoga contributed to increased wellbeing and reduced occupational and life stress by paying attention to the experiences of 13 yoga practitioners who practiced yoga at work, home, or elsewhere. In line with the Salutogenic perspective on health, we viewed our findings as attempts at regaining balance or homeostasis. We found that practicing yoga was experienced to have positive impacts on health and wellbeing to varying degrees for all participants in the present study. Yoga was experienced as providing increased wellbeing by making people feel more self-aware, energized, lighthearted, more positive, better at self-care, more aware of their needs and limitations, and more inward-focused. Moreover, yoga was experienced as contributing to building self-confidence, a sense of mastery, and focus. Thus, it was not surprising that most of the informants in this study expressed that they were dedicated to continuing with yoga, as they perceived yoga as beneficial for them in their everyday lives, both at work and beyond.

Furthermore, practicing yoga was experienced as contributing to the interviewees coping with stress and reducing stress: by going more inwards, feeling calmer, happier, and more satisfied, being less sensitive to others’ expectations, being more peaceful, having less stiffness in the body, having less muscle tension, and having less worry regarding everything they feel they “have to do.” As described in the discussion of results, our findings confirm and concretize the meta-analysis by numerous authors on yoga, stress, and wellbeing, which found that most studies on yoga and occupational yoga found a significant increase in wellbeing as well as a reduction in stress after yoga interventions((12, 13, 30, 36, 75, 82). In our study, the informants interpreted their yoga experiences as facilitating that they were on a path of positive self-development (or even self-actualization).

As a framework for our study, we also find the Salutogenic perspective valuable in realizing that experiencing wellbeing or stress are not separate categories but at different ends of a continuum. It also seemed that practicing yoga was providing our interviewees with a greater sense of coherence (SOC), which, as discussed earlier, is a central concept in the Salutogenic perspective (see (58)). Yoga seemed to make the informants’ lives and work lives more meaningful in the sense that they became more in touch with their inner selves and achieved more presence in their lives. Yoga contributed to comprehensibility by increasing our interviewees’ sense of focus, calm, and balance. Informants in our study also expressed that yoga contributed to a sense of mastery, which made it easier to cope with stress and manage their work (lives). All of these factors contributed positively to their (mental) health and wellbeing.

Especially the yogic breath, or Pranayama, was emphasized as contributing to reducing stress, and as something that the informants could use to calm down and reduce stress (cf. (24, 45)). Many of our participants used breathing exercises or practiced deep breathing consciously during the workday to find calmness in between meetings, prepare for performance, and become peaceful rather than overwhelmed. Sometimes breathing is used together with meditation and positive self-talk to become relaxed and focused rather than stressed. It seemed that practicing yoga would strengthen salutary factors, as discussed above, factors that actively contributed to health and wellbeing, including a greater sense of coherence (SOC). Also related to the informants’ experiences of the role of breathing, it appeared that the experiences of wellbeing and stress were experienced along a continuum, like it is claimed in the salutogenic perspective of health, rather than as either-or.

Naturally, contextual factors played a role in how practicing yoga was experienced. The context for doing yoga was also significant, such as the time, the instructor, and the workplace setting. Morning yoga was seen to facilitate a good start to the working day, but yoga at the end of the workday or after work would allow them to destress and reenergize, and often make them more ready for the next workday. The yoga instructor or teacher played an important role in the experience of the interviewees, for example, through the pace of the yoga practice they offered, being trustworthy, and as a motivator and guide. It seemed particularly important that the yoga teacher showed interest in understanding the participants. Furthermore, it was perceived as important that the teachers were able to see each of the participants and that they were caring, rather than too strict. The participants in our study varied in where they practiced yoga, but especially those who were offered yoga at their workplace felt gratitude for being offered yoga at their job. Several of the informants expressed that yoga made them happier and more productive, expressing that they were rounder, more relaxed, less irritated, and better and more patient with other people. All of these are qualities that facilitate good cooperation in the workplace and a better working environment. This is good news for contemporary workplaces.

Our study supports what is well established in the research literature: that yoga is an efficient way to manage stress, even in workplaces (51, 52, 75, 83). Our research findings also support the increasing research focus on wellbeing in the workplace (44, 56). One of the advantages of qualitative studies like ours is that they illustrate the elements that constitute to the experiences of less stress and increased wellbeing for different individuals.

Still, workplaces must not use yoga as a “resting pillow.” Work overload and stressful working conditions need to be reduced in many professions. As pointed out by the Norwegian Labor Inspection Authority, factors like too long workdays, discrepancy between tasks and resources, contradictory demands, tight deadlines, unclear role expectations and communication, bad change processes, bullying at work, and insufficient social support from leaders and colleagues are creating stressful workplaces. Over time, such stress factors and workplaces can be detrimental to human health, both mentally and physically (see text footnote 1). After the COVID-19 pandemic, there has been an increased focus on reducing stress in workplaces. There has also been more attention to the distinction between task-oriented and more space-bound employees, with their different kinds of work-related stress.

Regardless of professions and workplaces, optimal working conditions are needed for all employees, while also aiming to strengthen the employees, or “the human factor,” through, for example, occupational yoga. As research on yoga in life and work–life indicates, yoga can offer tools to increase wellbeing and enable employees to become calmer and more relaxed, less stressed, and better able to deal with existing life and work challenges (33, 48, 50). If yoga is offered in the workplace, there is also a need to understand better how and why yoga facilitates mental and physical health and wellbeing. In our study, it seemed that the ability to go inwards created more awareness, feeling calm, energized, focused, and more able, both mentally and physically. Necessary self-care became more important than living up to often contradictory expectations and becoming stressed by external pressure. Such needs for relaxation and finding calm and peace of mind are also emphasized by others (27, 76). In our study, the breath was mentioned as especially useful for calming down rather than being stressed.

For future research, there is also a need to focus on how different kinds of yoga can contribute, for different people, genders, ages, and professions, to workplace wellbeing programs. Yoga seems to influence both the HPA (hypothalamic–pituitary–adrenal axis) and the SAM (sympathetic–adrenal–medullary system) (84). Mechanisms include a reduction in the activation or response of the sympathetic nervous system (SNS) and the HPA axis, thus preventing the release of stress hormones like cortisol and catecholamines (34, 41, 85). However, focusing on wellbeing and health needs to be more in focus, as they are on the other side of the stress–wellbeing continuum. Thus, we suggest that a salutogenic perspective should be part of the analysis, focusing on factors that can contribute to health promotion and increased wellbeing. This can complement the focus on stress reduction through relaxation and learning how to cope with stress. The role of Pranayama, or yogic breathing, is also worth exploring further, both when developing interventions and for further research. Contextual factors that may influence the significance of yoga in occupational locations and beyond are also worth examining further.

## Data availability statement

The dataset presented in this article is not readily available because it was stored for a limited time.

## Ethics statement

The studies involving humans were approved by this study was reviewed and approved by NSD.no, the Norwegian council for approving research. The studies were conducted in accordance with the local legislation and institutional requirements. The participants provided their written informed consent to participate in this study.

## Author contributions

IH: Investigation, Writing – original draft, Writing – review & editing. ØH: Writing – review & editing.

## Footnotes

^1^ https://www.arbeidstilsynet.no/tema/stress/

^2^ https://arbeidstilsynet.no/nyheter/ny-arbeidslivsrapport-mer-hjemmekontor-og-teknostress/

^3^ https://www.who.int/about/accountability/governance/constitution

^4^ https://sdgs.un.org/goals/goal3
